# Impaired Cardiac Function in Patients with Multiple Sclerosis by Comparison with Normal Subjects

**DOI:** 10.1038/s41598-018-21599-0

**Published:** 2018-02-19

**Authors:** Raluca Ileana Mincu, Stefania Lucia Magda, Sorina Mihaila, Maria Florescu, Diana Janina Mihalcea, Andreea Velcea, Adela Chiru, Cristina Tiu, Bogdan Ovidiu Popescu, Mircea Cinteza, Dragos Vinereanu

**Affiliations:** 1 University of Medicine and Pharmacy Carol Davila, Bucharest, Romania; 2 University and Emergency Hospital, Bucharest, Romania; 3 Colentina Clinical Hospital, Bucharest, Romania

## Abstract

Multiple sclerosis (MS), neurologic disease affecting young population, may cause cardiovascular dysfunction, due to autonomous nervous dysfunction, physical invalidity, increased oxidative stress, and systemic inflammatory status. However, cardiovascular function is rarely evaluated in these patients. We assessed left and right ventricular (LV and RV) function by 2D, 3D, tissue Doppler, and speckle tracking echocardiography, and vascular function by remodeling, stiffness, and endothelial dysfunction parameters in patients with MS, compared to control subjects. 103 subjects (35 ± 10 years,70 women) were studied: 67 patients with MS and 36 control subjects. Patients with MS had decreased LV systolic function, confirmed by lower 2D and 3D ejection fraction, mitral annular plane systolic excursion, longitudinal myocardial systolic velocities, and 2D and 3D global longitudinal strain. The RV function was also decreased, as demonstrated by lower fractional area change, tricuspid annular plane systolic excursion, longitudinal systolic velocities, and longitudinal strain. Additionally, LV diastolic and left atrial (LA)  function were decreased compared to controls. The parameters of arterial and endothelial function were similar between groups. Patients with MS have impaired biventricular function by comparison with normal subjects, with reduced LA function, but normal arterial and endothelial function. The noninvasive echocardiographic techniques might help to determine patients with MS at risk of developing cardiovascular dysfunction.

## Introduction

Multiple sclerosis (MS) is a chronic neurological condition, characterized by recurrent episodes of inflammation and demyelinisation of the central nervous system, that determine axonal degeneration and lead to irreversible progressive invalidity^1^. MS is the main cause of non-traumatic neurological invalidity in young and middle-aged populations, affecting women two times more frequently than men^2^.

The main mechanisms of impaired cardiovascular (CV) function in patients with MS are the cardiomyocite structure alteration, the CV autonomous nervous system dysfunction, physical invalidity, oxidative stress, endothelial dysfunction, and the presence of associated cardiovascular risk factors^3^. Patients with MS have a higher mortality rate compared to the general population, and this might be related to an higher incidence of CV disease^4,5^. However, cardiovascular function assessment is rarely performed in these patients. Most studies focus on the cardiotoxicity of mitoxantrone, which is nowadays a second line treatment^6,7^. Additionally, the majority of these studies assess conventional 2D-echocardiography and tissue Doppler parameters, however, the new techniques, such as speckle tracking echocardiography or real-time 3D-echocardiography, although simple, rapid, and effective in providing early diagnosis of myocardial dysfunction are not used^8,9^.

There is little knowledge about the cardiovascular function in MS, and subsequent research is required to improve the understanding of these mechanisms and to develop management algorithms capable to detect and prevent CV adverse events in this population. Therefore, our objective was to extensively assess cardiac and arterial function in patients with MS, compared to control subjects, in order to provide a pilot model for the early detection of CV dysfunction in these group of young patients.

## Results

### Study population

We prospectively enrolled 103 subjects (mean age 35 ± 10 years old, 70 women), divided in two groups: the MS group with 67 subjects with confirmed MS, and the control group with 36 control subjects. Within the MS group, 35 patients were already treated for more than 6 months with interferon or glatiramer acetate, while 32 patients were newly diagnosed and free of immunomodulatory treatment. Mean duration of MS was 5.2 ± 5.0 years. The two study groups had similar mean age and gender distribution, with an equal incidence of CV risk factors, such as arterial hypertension, dyslipidemia, smoking, and obesity (Table 1). The Expanded Disability Status Scale (EDSS) score in the MS group was 2.5 ± 1.5, showing mild disability.

**Table 1:** General characteristics of patients with multiple sclerosis and control subjects; data are expressed as mean ± SD or percentages.

Variables	Multiple Sclerosis	Control Subjects	P-value
Age (years)	36 ± 10	34 ± 10	NS
Female sex (%)	68	67	NS
Body mass index (kg/m^2^)	23.5 ± 3.8	23.5 ± 3.9	NS
Body surface area (m^2^)	1.7 ± 0.1	1.7 ± 0.2	NS
Systolic blood pressure (mmHg)	117 ± 8	117 ± 7	NS
Diastolic blood pressure (mmHg)	75 ± 5	73 ± 6	NS
Heart rate (beats/min)	76 ± 10	75 ± 7	NS
Dyslipidemia (%)	16	16	NS
Smoking (%)	28	17	NS

### Left ventricular (LV) and right ventricular (RV) systolic function

**Figure 1:**
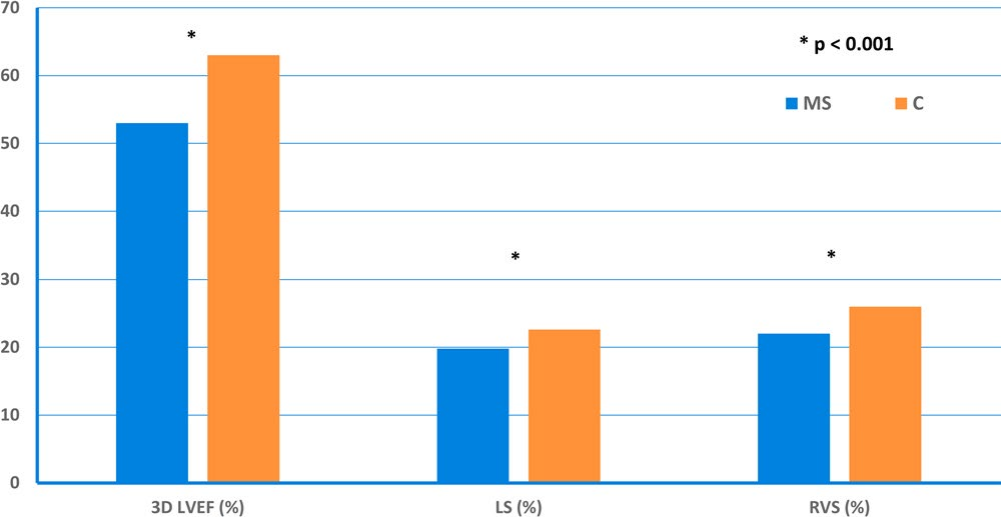
The 3-dimensional left ventricular ejection fraction (3D LVEF), 2-dimensional longitudinal strain (LS) and right ventricular strain (RVS) were lower in patients with multiple sclerosis (MS) compared to control subjects (C).

Patients with MS had decreased LV systolic function compared to control subjects, as showed by lower 2D left ventricular ejection fraction (LVEF), lower 3D LVEF, lower mitral annular plane systolic excursion (MAPSE), lower “online” longitudinal myocardial systolic velocity (S’), lower global longitudinal strain (LS), and lower 3D LS (Table 2  and Figure 1). Meanwhile, patients with MS had decreased RV systolic function compared to control subjects, as showed by lower fractional area change (FAC), lower tricuspid annular plane systolic excursion (TAPSE), affected RV myocardial performance index (RVMPI), lower peak tricuspid annular systolic velocity (RV S’), and lower RV strain. Additionally, patients with MS had higher systolic pulmonary artery pressure (sPAP) than the control subjects (Table 2 and Figure 1).

**Table 2:** Left ventricular and right ventricular systolic function, assessed by echocardiography, of patients with multiple sclerosis and control subjects. Data are expressed as mean ± SD.

Echo Parameters	Multiple Sclerosis	Control Subjects	P-value
LVEDD (mm)	46 ± 6	45 ± 4	NS
LVESD (mm)	27 ± 5	28 ± 5	NS
LVEDV (ml)	91 ± 22	96 ± 22	NS
LVESV (ml)	41 ± 10	34 ± 11	0.005
2D LVEF (%)	55 ± 5	65 ± 5	<0.001
LV mass index (g/m^2^)	81 ± 30	67 ± 15	0.021
MAPSE (mm)	14 ± 2	17 ± 1	<0.001
S’ (cm/s)	6.0 ± 1.0	7.4 ± 1.0	<0.001
S’ online (cm/s)	9.2 ± 2.3	10.3 ± 2.3	0.029
LS (%)	−19.8 ± 2.2	−22.6 ± 1.7	0.000
LSR (1/s)	−1.1 ± 0.5	−1.2 ± 0.1	0.26
RS (%)	46 ± 14	50 ± 13	NS
RSR (1/s)	2.7 ± 1	2.5 ± 0.8	NS
CS (%)	−22 ± 5	−20 ± 9	NS
CSR (1/s)	−5.3 ± 26	−1.6 ± 0.9	NS
3D LVEDV (ml)	83 ± 24	78 ± 23	NS
3D LVESV (ml)	39 ± 13	28 ± 9	0.006
3D LVEF (%)	53 ± 6	63 ± 3	<0.001
CO (ml/min/m^2^)	3.1 ± 1.0	3.5 ± 0.8	NS
3D LS (%)	−15.0 ± 3.0	−20.0 ± 1.7	<0.001
RVEDD (mm)	30 ± 3	30 ± 3	NS
FAC (%)	42 ± 7	53 ± 8	<0.001
TAPSE (mm)	22 ± 2	26 ± 2	<0.001
RV S’ (cm/s)	12.9 ± 2.0	14.7 ± 2.0	0.001
RVMPI	0.56 ± 0.10	0.40 ± 0.06	<0.001
RVS (%)	−22.5 ± 3.3	−26 ± 4	<0.001
RVSR (1/s)	−1.4 ± 0.3	−1.3 ± 0.8	NS
sPAP (mmHg)	26 ± 9	14 ± 6	<0.001

LV = left ventricle; LVEDD = left ventricular end-diastolic diameter; LVESD = left ventricular end-systolic diameter; LVEDV = left ventricular end-diastolic volume; LVESV = left ventricular end-systolic volume; 2D LVEF = 2-dimensional left ventricular ejection fraction; MAPSE = mitral annular plane systolic excursion; S^’^ = 6-site averaged longitudinal myocardial offline systolic velocity; S’ online = online longitudinal myocardial systolic velocity, calculated as mean of the systolic velocities of the lateral and medial mitral annulus; LS = longitudinal strain; LSR = longitudinal strain rate; RS = radial strain; RSR = radial strain rate; CS = circumferential strain; CSR = circumferential strain rate; 3D LVEDV = 3-dimensional  left ventricular end-diastolic volume; 3D  LVESV = 3-dimensional  left ventricular end-systolic volume;  3D LVEF = 3-dimensional left ventricular ejection fraction; CO = cardiac output; 3D LS = 3-dimensional longitudinal strain; RVEDD = right ventricular end-diastolic diameter; FAC = fractional area change; TAPSE = tricuspid annular plane systolic excursion; RV S^’^ = peak tricuspid annular systolic velocity measured online; RVMPI = right ventricular myocardial performance index; RVS = right ventricular strain; RVSR = right ventricular strain rate; sPAP = systolic pulmonary artery pressure.

### LV diastolic function

Patients with MS had impaired LV diastolic function, compared to control subjects, as showed by lower early (E) and late (A) diastolic velocities, lower flow propagation velocities (FPV), and longer isovolumetric relaxation time (IVRT). Meanwhile, in patients with MS, early “online” longitudinal myocardial diastolic velocity (E’) was lower than in control subjects, although the E/E’ ratio was similar between groups. In patients with MS, the untwist rate (UTR) was reduced compared to control subjects, as well as the left atrial systolic  longitudinal strain (LA SLS) (Table 3).

**Table 3:** Left ventricular diastolic function, assessed by echocardiography, of patients with multiple sclerosis and control subjects. Data are expressed as mean ± SD.

Echo Parameters	Multiple Sclerosis	Control Subjects	P-value
E velocity (cm/s)	74 ± 15	85 ± 16	0.002
A velocity (cm/s)	53 ± 11	60 ± 13	0.020
E/A	1.41 ± 0.30	1.45 ± 0.40	NS
LA volume index (mm^3^/m^2^)	24 ± 7	27 ± 8	NS
FPV (cm/sec)	47 ± 11	70 ± 21	<0.001
IVRT (sec)	93 ± 11	69 ± 11	<0.001
E/FPV	1.67 ± 0.40	1.32 ± 0.30	0.002
E’(cm/s)	9.3 ± 2.0	10.6 ± 2.0	0.010
E’ online (cm/s)	14 ± 4	16 ± 3	0.05
E/E’	6.0 ± 1.4	6.0 ± 1.3	NS
UTR (°/s)	−100 ± 36	−129 ± 33	0.003
LA SLS (%)	−12 ± 3	−14 ± 2	0.027

E = early diastolic velocity; A = late diastolic velocity; LA = left atrial; FPV = flow propagation velocity; IVRT = isovolumetric relaxation time; E^’^ = 6-site averaged longitudinal myocardial offline diastolic velocity; E’ online = online longitudinal myocardial diastolic velocity; UTR = untwist rate, LA SLS = left atrial systolic longitudinal strain.

### Arterial function

Arterial remodeling, assessed by intima media thickness (IMT), was similar between the groups. Arterial stiffness, assessed by Peterson elastic module Ep, β index, augmentation index (Aix), arterial compliance, and pulse wave velocity carotid-femoral (PWV CF), and endothelial function, assessed by flow mediated dilation (FMD), were also similar between groups (Table 4).

**Table 4:** Arterial function of patients with multiple sclerosis and control subjects. Data are expressed as mean ± SD.

Parameter	Multiple sclerosis	Control Subjects	P-value
IMT (mm)	0.5 ± 0.1	0.6 ± 0.1	NS
Ep (kPa)	70 ± 34	61 ± 30	NS
β index	5.5 ± 2.5	5.3 ± 2.1	NS
AiX (%)	15.8 ± 7.24	3.9 ± 3	NS
AC (cm^2^/mmHg)	1.1 ± 0.3	1 ± 0.4	NS
PWV CF (m/s)	7.32 ± 1.47	6.34 ± 1.68	NS
FMD (%)	15.29 ± 9.44	16.72 ± 11.46	NS

IMT = intima media thickness; Ep = Peterson’s elastic modulus; AiX = augmentation index; AC = arterial compliance; PWV CF = pulse wave velocity carotid-femoral; FMD = flow mediated dilation.

### Biomarkers

The troponin I value, as a marker of myocardial ischemia, was similar between the two groups: 0.0007 ± 0.001 ng/ml in the MS group and 0.0012 ± 0.002 ng/dl in the control group. The N-terminal pro B-type natriuretic peptide (NT-pro BNP), as a marker of heart failure, was also similar between groups: 35 ± 26 pg/ml in the MS group and 37 ± 32 pg/ml in the control group.

### Subgroups analysis

We performed a subgroup analysis, by dividing patients with MS into newly diagnosed MS patients (MS_1_ = 32 patients) and treated MS patients (MS_2_ = 35 patients). There were no significant differences between the two subgroups for any of the echocardiographic parameters, but each of them was different from the control subjects (Supplementary Table 1).

## Discussion

Our study provides a comprehensive evaluation of cardiovascular function in patients with MS compared to matched control subjects. We demonstrated that patients with MS, by comparison with control subjects, have impaired LV systolic and diastolic function, with reduced LA function, impaired RV function with increased systolic pulmonary artery pressure, but similar arterial and endothelial function. Meanwhile, biomarkers of myocardial ischemia and heart failure were similar between groups. These findings are significant for the daily clinical practice, by emphasizing that this young and active population should receive the best standard of care from a multidisciplinary team, including a cardiologist. Based on these results, we suggest the use of the new echocardiographic techniques for the early detection of cardiovascular dysfunction in patients with MS, because they are harmless, inexpensive, and largely available. The advance in the therapy of MS significantly improves survival and morbidity, but we should not allow this effect to be diminished by the cardiovascular morbidity^10^. Our exploratory study needs further confirmation before being implementing by guidelines in patients with MS, however, it opens important premises for further research and innovation in the new field of neuro-cardiology.

Patients with MS have a lower life expectancy than normal population, but detailed data on mortality in MS are limited^11^. Recent population studies^12^ showed that the median survival time from onset of disease was approximately 10 years shorter for patients with MS, than for the age‐matched subjects, and MS was associated with an almost threefold increase in the risk of death. This increase in mortality might be also related to cardiovascular dysfunction^13^, but most of the studies did not differentiate between various types of causes of death. Two large studies revealed that MS was associated with an increased risk of hospital admission for ischemic stroke, myocardial infarction, and heart failure in the first year of diagnosis, and that the increased risk for heart failure persisted over time, suggesting that early diagnosis of cardiac dysfunction in patients with MS might be crucial in order to start preventive measures^14,15^. The detailed mechanisms of cardiovascular dysfunction in patients with MS are not completely elucidated. Involvement of myocyte structure was showed in neuromuscular disorders, such as muscular dystrophies and myofibrillar, congenital, and metabolic myopathies^16^. It was also showed that isoforms of mutated muscle proteins causing muscle disease are also expressed in the myocardium, and this might be the main mechanism responsible for the impairment of the cardiac function in our study^17^. Additionally, the mitochondrial dysfunction is present in the central nervous system, leading to disruption of myelin production, changes that could also be present in the cardiac cells and contribute to the impairment of the cardiac function in this population^18,19^.

Oxidative stress is increased several times in MS^20^ and, associated with vascular inflammation, it may lead to endothelial dysfunction, arterial remodeling and stiffness, and overt atherosclerosis^21^. However, in our study both endothelial and arterial functions were similar between patients with MS and control subjects, suggesting an intrinsic myocardial involvement in these patients. Other factors that might contribute to the cardiovascular dysfunction in patients with MS are related to different prevalence of the risk factors in the MS population, since smoking, dyslipidaemia, and lack of exercise were all proved to be more frequent in patients with MS^22,23^.

Initial studies using echocardiography focused on cardiotoxicity of mitoxantrone in patients with MS^6,7^. Concordant with our finding, they reported lower myocardial performance index and shorter isovolumic relaxation time in patients with MS, when compared to controls, but on a small number of patients. Another study investigated LV and RV function in patients with MS, compared to control subjects, and showed that LV ejection fraction was decreased in patients with MS^8^. However, they used only conventional echocardiography, although new techniques, such as 3D-echocardiography or speckle tracking, might have had a better accuracy in detecting subtle, subclinical changes of the myocardial function. In fact, there are no studies published so far, using assessment of myocardial function by 3D-echocardiography and speckle tracking in patients with MS. Moreover, in the previous studies using tissue Doppler, number of parameters assessed was limited^6–8^. To the best of our knowledge, our study is the first one which used such a comprehensive cardiovascular assessment in patients with MS. Our results might contribute to the development of new diagnostic tools and prognostic scores for the cardiovascular dysfunction in patients with MS. Their introduction into clinical practice will allow early diagnosis of cardiovascular dysfunction in patients with MS, leading to an optimization of cardiovascular prevention, and disease specific treatment. This is an important issue, because MS is the main cause of non-traumatic neurological morbidity and, therefore, optimization of the medical management of these young, socially and economically active patients represents a priority.

### Study limitations

The correct diagnosis of MS may be challenging, because the disease might be active long before the first clinical sign. The immunomodulatory treatment was heterogeneous in our treated patients, however, there were no differences between the treated and the untreated subgroups of patients with MS. This study was exploratory and needs further research in order to define the cut-off values of different echocardiographic parameters for the diagnosis of subclinical cardiac dysfunction in patients with MS.

## Conclusion

Patients with MS have impaired biventricular function by comparison with normal subjects, with reduced LA function, but normal arterial and endothelial function, suggesting an intrinsic myocardial disease. Use of noninvasive echocardiographic techniques might help to determine patients with MS at risk of developing cardiovascular dysfunction.

## Methods

### Study population

We prospectively enrolled patients with confirmed MS, diagnosed according to the revised McDonald’s criteria^24^, and control subjects matched for age, gender, and presence of cardiovascular risk factors. Inclusion criteria for patients with MS were: (1) age between 18 and 65 years; (2) patients with confirmed MS diagnosis, both newly diagnosed or already under immunomodulatory treatment; and (3) informed consent signed. Exclusion criteria were: (1) MS treated with mitoxantrone; (2) patients with known cardiovascular disease; (3) presence of other neurological conditions; (4) any renal, pulmonary, hepatic or hematological disease; (5) diabetes mellitus; (6) any degree of arterial hypertension; (7) pregnancy; (8) difficult acoustic window. The study protocol was approved by the Local Ethic Committee of the University Emergency Hospital Bucharest (approval number 5/2013), and registered on clinicaltrials.org under the identifier NCT03001284. All subjects gave their informed consent and the protocol conforms to the ethical guidelines of the 1975 Declaration of Helsinki, as reflected in a priori approval by our institution’s human research committee. Blood pressure, height, and weight were measured before the echocardiographic examination. Body mass index and body surface area were calculated by Du Bois formula. Following biomarkers were measured: troponin I as a marker of myocardial ischemia and NT-pro BNP as a marker of heart failure. In patients with MS, the invalidity level was calculated using the EDSS score^25^.

### Echocardiography

All subjects underwent comprehensive echocardiographic examination using a commercially available ultrasound machine (Vivid E9, GE Vingmed Ultrasound, Horten, Norway), equipped with a M4S probe and a 3D probe. Standard images were obtained from parasternal long- and short-axis views, and from apical views. Special acquisitions were performed for tissue Doppler, speckle tracking, and 3D-echocardiography. Data were analyzed offline, using a dedicated software package (EchoPac 9.0.1 for PC; GE Medical System). Electrocardiogram was recorded simultaneously.

*Conventional echocardiography* consisted of M-mode, 2D, and Doppler blood flow measurements, performed according to the current guidelines^26^. M-mode was used to measure systolic and diastolic thickness of the posterior and septal walls, and left ventricular, systolic and diastolic diameters. We calculated LV volumes and ejection fraction by Simpson’s method, and LV mass and LV mass indexed to body surface area estimated by LV cavity dimension and wall thickness at end-diastole. Mitral annular plane systolic excursion (MAPSE) was calculated from the mean of the lateral and medial annular excursions. Diastolic function was assessed by PW Doppler of the transmitral flow, and E/A ratio was calculated. LV inflow was recorded by color M-mode, flow propagation velocity (FPV) was measured, and E/FPV was calculated^27^. Right ventricular diameters, area and area change, and tricuspid annular plane systolic excursion (TAPSE) were measured according to the current guidelines^28^. Systolic pulmonary artery pressure (sPAP) was estimated from the sum of RV systolic pressure estimated from tricuspid regurgitation velocity and right atrial pressure estimated from inferior vena cava and its collapsibility^28^.

*Tissue Doppler* was used to calculate the peak longitudinal, systolic and diastolic myocardial velocities in the offline analysis (S’ and E’, respectively), averaged from six basal segments of the left ventricle, from 2-, 3-, and 4-chamber views. The online S’ and E’ were measured; online E/E’ ratio was calculated as a marker of ventricular filling^29^. Right ventricular myocardial performance index (MPI) was calculated from the online pulsed tissue Doppler curves of the tricuspid annulus, using the formula MPI = (TCO −  ET)/ET, where TCO is tricuspid valve closure-to-opening time and ET is ejection time^28^.

*Speckle tracking* was used to assess systolic myocardial deformation in three directions: longitudinal, circumferential, and radial. Peak systolic longitudinal strain (LS) and strain rate (LSR) were calculated as a mean of 18 ventricular segments from the apical views, while peak circumferential strain (CS) and strain rate (CSR), and peak radial strain (RS) and strain rate (RSR) were calculated from 6 ventricular segments from the short-axis view at the level of the papillary muscles^9,30^. Right ventricular longitudinal strain and strain rate were determined using the same software originally developed for LV^28^. LV twist, defined as absolute apex-to-base gradient rotation along the longitudinal axis of the LV, expressed in degrees, was assessed at the short-axis view at the mitral valve level, and the short-axis view at the apical level. Apical and basal rotations were measured, and LV twist was calculated as RotA-RotB. LV torsion was obtained by dividing LV twist to LV end-diastolic longitudinal length (degree/cm)^31,32^. LV untwist, a parameter of LV diastolic function, was measured, as well as untwist rate (degree/seconds), as the mean difference between the untwist curves. Finally, left and right atrial functions were also quantified^33^.

*Real-time 3D-echocardiography* was used to assess left ventricular end-diastolic and end-systolic volumes, ejection fraction, cardiac output, left ventricular mass, and left ventricular strain, using the software 4DLVQ (“4D left ventricle quantification”). The 3 apical views were corrected to display standard 2-, 3-, and 4-chamber views, eliminating the foreshortening. The end-diastolic frame was automatically detected from the electrocardiogram, and three points (two for the mitral plane and one for the left ventricular apex) were defined manually for each of the three views, and the endocardial border was automatically traced. Then, the endocardial border was adjusted to obtain the optimal tracing. Time-volume curves were generated^34,35^.

### Arterial function

Subjects were assessed after 10 minutes of rest in the supine position, in a constant room temperature, after 8–12 hours of fasting. Smoking, ingestion of vasoactive medication, caffeine and alcohol was not permitted 4–6 hours before the examination^36^. Blood pressure and heart rate were monitored. Intima-media thickness (IMT) was measured at the right common carotid artery, 1 cm below bifurcation, using Aloka Prosound α10 ultrasound machine (Japan), with a 7.5 MHz linear array probe. The far wall was used to measure mean IMT, and the final value was averaged from 3 different measurements^37^. The arterial stiffness was analyzed at the level of the right common carotid artery^38^. The machine allows measurement of flow velocity simultaneously with the artery diameter, using high-resolution online tracking wall (“E-tracking technology”). Arterial pressure waveforms were obtained, and 5 beats were signal-averaged to give a single waveform of diameter and velocity. Using this waveform, the following parameters were calculated: Peterson’s pressure-strain elastic modulus (Ep), β stiffness index, and local arterial compliance (AC). Augmentation index (Aix) was derived from the diameter-derived pressure waveform. Carotid-femoral pulse wave velocity was measured by Complior System (Artech Medical, Pantin, France)^39^. Endothelial function was analyzed by flow mediated dilation, at the level of the right brachial artery, and expressed as a percentage of the baseline diameter^40,41^.

### Statistical analysis

Statistical analysis was performed with SPSS software version 20 (IBM Statistics 20). Continuous variables were reported as mean and standard deviation (SD). Categorical variables were reported as percentages. Independent samples T test was used to compare continuous variables, while chi-square test was used to compare categorical variables. Pearson’s correlation was used for the association between variables. Differences with p-values ≤ 0.05 (2-sided) were considered statistically significant. Good reproducibility and repeatability of vascular function parameters were reported previously in our laboratory in 20 consecutive patients by two observers with similar experience for IMT, PWV, and parameters of arterial stiffness^41^.

## Electronic supplementary material

Supplementary Information
