# Supplementary material for: Impaired Cardiac Function in Patients with Multiple Sclerosis by Comparison with Normal Subjects
Source: Sci Rep. 2018 Feb 19;8:3300. doi: 10.1038/s41598-018-21599-0 (PMC5818507; doi:10.1038/s41598-018-21599-0)
Supplement: Supplementary file 1 — Supplementary Information [file 41598_2018_21599_MOESM1_ESM.doc]

**Impaired Cardiac Function in Patients with Multiple Sclerosis by Comparison with Normal Subjects**

Raluca Ileana Mincu, MD, PhD^a,b^; Stefania Lucia Magda, MD, PhD^b^; Sorina Mihaila, MD, PhD^a,b^; Maria Florescu, MD, PhD^a,b^; Diana Janina Mihalcea, MD^a^; Andreea Velcea, MD^a^; Adela Chiru, MD^c^; Cristina Tiu, MD, PhD^a,b^; Bogdan Ovidiu Popescu, MD, PhD^a,c^; Mircea Cinteza, MD, PhD^a,b^; Dragos Vinereanu MD, PhD, FESC, FRCP^a,b*^

^a^University of Medicine and Pharmacy Carol Davila, Bucharest, Romania.

^b^University and Emergency Hospital, Bucharest, Romania.

^c^Colentina Clinical Hospital, Bucharest, Romania.

*Corresponding author:

Professor Dragos Vinereanu

University of Medicine and Pharmacy Carol Davila Bucharest

University and Emergency Hospital – Cardiology

Splaiul Independentei 169, 050098 Bucharest, Romania

Tel.: +40722670013 Fax: +40216012345

Email: [vinereanu@gmail.com](mailto:vinereanu@gmail.com)

Supplementary Table 1. The subgroup analysis of systolic LV and RV echocardiography parameters. We compared the newly diagnosed MS patients (MS1) with treated MS patients (MS2) and with the control patients (C).

**p value MS 1 vs C and MS 2 vs C < 0.001; p MS 1 vs MS 2 NS**

| **Parameter** | **MS 1** | **MS 2** | **C** |
| --- | --- | --- | --- |
| **2D LVEF (%)** | 55 ± 6 | 55 ± 4 | 65 ± 5 |
| **3D LVEF (%)** | 53 ± 7 | 53 ± 5 | 63 ± 3 |
| **S’ (cm/s)** | 5.7 ± 0.9 | 6 ± 1 | 7.4 ± 1 |
| **LS (%)** | -19 ± 2.5 | -20 ± 1.9 | -22.6 ± 1.7 |
| **3D LS(%)** | -15.1 ± 2.7 | -16.4 ± 3.3 | -20 ± 1.7 |
| **FAC (%)** | 41.7 ±7.9 | 42.0 ± 7 | 53.0 ± 8 |
| **TAPSE (mm)** | 22 ± 2 | 22 ± 2.4 | 26 ± 2 |
| **RVMPI** | 0.57 ± 0.1 | 0.55 ± 0.1 | 0.4 ± 0.06 |
| **RVS (%)** | -22.1 ± 3.3 | -22 ± 3.0 | -26 ± 4 |
| **sPAP (mmHg)** | 25 ± 7 | 7.7 ± 1.8 | 14.0 ± 6 |

2D LVEF = 2-dimensional left ventricular ejection fraction; 3D LVEF = 3-dimensional left ventricular ejection fraction; S’= 6-site averaged longitudinal myocardial offline systolic velocity; LS = longitudinal strain; 3D LS = 3-dimensional longitudinal strain; FAC = fractional area change; TAPSE = tricuspid annular plane systolic excursion; RVMPI = right ventricular myocardial performance index; RVS = right ventricular strain; sPAP= systolic pulmonary artery pressure.
